# The when and how of male circumcision and the risk of HIV: a retrospective cross-sectional analysis of two HIV surveys from Guinea-Bissau

**DOI:** 10.11604/pamj.2016.23.21.7797

**Published:** 2016-02-01

**Authors:** Dlama Nggida Rasmussen, Christian Wejse, Olav Larsen, Zacarias Da Silva, Peter Aaby, Morten Sodemann

**Affiliations:** 1 Bandim Health Project, Indepth Network, Apartado 861, 1004 Bissau Codex, Bissau, Guinea-Bissau; 2 Department of Infectious Diseases, Odense University Hospital, DK-5000 Odense, Denmark; 3 Center for Global Health, Institute of Clinical Research, University of Southern Denmark, DK-5000 Odense, Denmark; 4 Department of Infectious Diseases, Aarhus University Hospital, DK-8200 Aarhus, Denmark; 5 Center for Global Health, Department of Public Health, Aarhus University, DK-8000 Aarhus, Denmark; 6 Statens Serums Institute, DK-2300 Copenhagen, Denmark

**Keywords:** Africa, circumcision, HIV/AIDS, HIV prevention, risk behaviour

## Abstract

**Introduction:**

Male circumcision (MC) reduces the risk of HIV, and this risk reduction may be modified by socio-cultural factors such as the timing and method (medical and traditional) of circumcision. Understanding regional variations in circumcision practices and their relationship to HIV is crucial and can increase insight into the HIV epidemic in Africa.

**Methods:**

We used data from two retrospective HIV surveys conducted in Guinea-Bissau from 1993 to 1996 (1996 cohort) and from 2004 to 2007 (2006 cohort). Multivariate logistical models were used to investigate the relationships between HIV risk and circumcision status, timing, method of circumcision, and socio-demographic factors.

**Results:**

MC was protective against HIV infection in both cohorts, with adjusted odds ratios (AORs) of 0.28 (95% CI 0.12-0.66) and 0.30 (95% CI 0.09-0.93), respectively. We observed that post-pubertal (≥13 years) circumcision provided the highest level of HIV risk reduction in both cohorts compared to non-circumcised. However, the difference between pre-pubertal (≤12 years) and post-pubertal (≥13 years) circumcision was not significant in the multivariate analysis. Seventy-six percent (678/888) of circumcised males in the 2006 cohort were circumcised traditionally, and 7.7% of those males were HIV-infected compared to 1.9% of males circumcised medically, with AOR of 2.7 (95% CI 0.91-8.12).

**Conclusion:**

MC is highly prevalent in Guinea-Bissau, but ethnic variations in method and timing may affect its protection against HIV. Our findings suggest that sexual risk behaviour and traditional circumcision may increases HIV risk. The relationship between circumcision age, sexual behaviour and HIV status remains unclear and warrants further research.

## Introduction

The HIV epidemic in Guinea-Bissau has changed drastically over the past 20 years. Guinea-Bissau has had the highest prevalence of HIV-2 in West Africa for many years, while HIV-1 was absent only three decades ago. In urban Guinea-Bissau, the HIV-1 prevalence has risen from 2.3% in 1996 to 4.6% in 2006, while HIV-2 has decreased from 7.4% to 4.4% in the same period [1]. Guinea-Bissau is affected by a generalised epidemic of HIV-1 and HIV-2 [2]. It is currently estimated that between 3.7-5.8% of the country's adult population is infected with HIV [3, 4]. This prevalence is disturbingly high compared to neighbouring countries such as Senegal and Guinea Conakry, which both have HIV prevalence below 2% [4]. The difference in circumcision prevalence has repeatedly been suggested as one of the main causes for the evident contrast between high HIV prevalent countries in Southern and East Africa versus lower prevalent countries in West Africa [5, 6]. Furthermore, studies have shown that HIV prevalence is generally lower in regions with high prevalence of traditionally practiced MC [5, 7]. Findings from previous observational studies and three randomised control trials have reported up to a 60% risk reduction of HIV infection during heterosexual intercourse after voluntary medical MC (VMMC) [8–11]. Furthermore, studies have found that the protective effect of VMMC is sustained after a period of 6 years [12, 13]. The World Health Organization and Joint United Nations Programme on AIDS have recommended adult male circumcision as a principal method of prevention of heterosexually acquired HIV infection in men from endemic HIV settings with low circumcision prevalence [14, 15]. While great financial and logistical efforts have been made in certain regions of Sub-Saharan African, little attention has been paid to male circumcision and the HIV epidemic in West Africa [16]. Furthermore, few studies have looked at the role of circumcision in communities with diverse ethnic and cultural backgrounds. In Guinea-Bissau, an estimated 80 percent of the male population is circumcised [14]. MC is predominantly conceived as a traditional rite of passage and is practiced among all of the various ethno-linguistic groups living in the country. Despite its frequency, there is considerable variation in the age of circumcision. While the Balanta ethnic group perform circumcision ceremonies in village groups every 4-6 years and usually at a later stage in life (approximately 40 years), other ethnic groups such as the Fula and Mandinga generally perform MC between 6 and 13 years of age [17]. Studies have shown that early circumcision (during infancy and pre-puberty) may provide partial protection against the Sexually transmitted infections (STIs) that are known to be more prevalent in uncircumcised men by the time the men become sexually active [18]. Meta-analyses based on observational studies have shown that MC protects against genital herpes, syphilis, oncogenic human papillomavirus and HIV [19–21]. The time in life at which male circumcision is performed may have the same implications for all STIs prevented by MC. An unpublished report regarding reproductive health among 1,500 adolescents in urban Guinea-Bissau found the median age of sexual debut was reported to be 16 years for males [22]. If a male is sexually active before he is circumcised, he may be exposed to a period of increased risk of HIV infection and other STIs [18]. Therefore, understanding regional variations in circumcision practices and circumcision age is important for targeting future interventions and provides insight regarding HIV epidemiology in Africa. In this study, we first assessed the prevalence of circumcision and the socio-demographic distribution of male circumcision in an urban population in Guinea-Bissau. Second, we investigated the relationship between circumcision status, age of circumcision, method of circumcision and HIV infection risk in our study population.

## Methods

This study was conducted using retrospective data from the Bandim Health Project (BHP), Bissau, Guinea-Bissau, in West Africa. The BHP is a Danish-Guinean Demographic Surveillance Site with a study-area consisting of 6 suburban districts in Guinea Bissau's capital, Bissau. Regular censuses and data collection are conducted at the study site. Furthermore, repeated HIV household surveys in the study area have been conducted since1987, with larger studies taking place every decade. Initially, houses were randomly selected and the adults living in 384 houses in the study area were subjected to subsequent follow-up every ten years in an open cohort. These studies have collected detailed socio-demographic data as well as data on HIV sero-status, male circumcision status and circumcision age. In the 2006 cohort, place of circumcision was also assessed. Circumcision status was ascertained by self-reporting, as previously described [1]. Data were collected using questionnaires administered by trained research assistants during door-to-door interviews.

**Figure 1:**
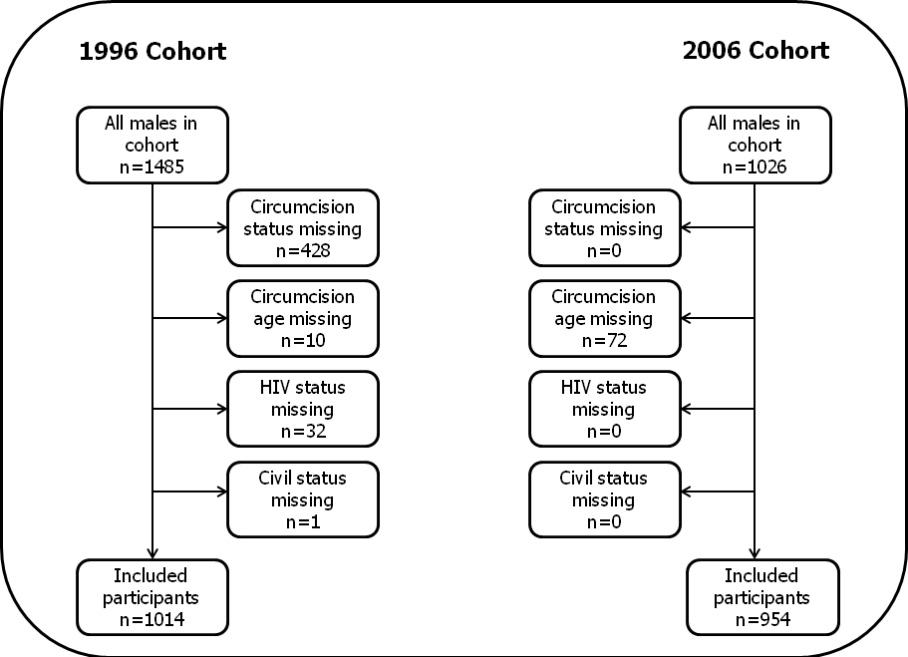
The inclusion process of males from the 1996 and 2006 cohort studies

**Study design:** For this retrospective cohort-based study, we used data from an HIV survey conducted from 1993 to 1996 and an HIV survey conducted from 2004 to 2007. We have denoted the two surveys as the “1996 cohort” and the “2006 cohort”, respectively. Both HIV surveys had identical designs and methods and were comprised of male and female individuals aged 14 years or older living in the BHP study area. Males with recorded data on HIV status, circumcision status, age of circumcision, ethnicity and civil status were included in this study. In the 1996 cohort, 68% of all males (1014/1485) were eligible for participation; 471 were excluded due to missing data on civil status, HIV status, circumcision status or circumcision age. In the 2006 cohort, 93% of all males (954/1026) were eligible for participation and 72 men were excluded due to missing data on circumcision age. Additionally, 231 men participated in both surveys of which 9 were HIV positive. The inclusion process is displayed in (Figure 1).

**Statistical analysis:** Data were entered using dBase V software. The analysis was carried out using STATA version 12.0 (STATA Corporation, College Station, Texas, USA). Median values are expressed with an inter quartile range (IQR). Pearson's χ^2^ test was used when comparing the prevalence of the population proportion. Univariate and multivariate logistical regression models were used to examine the associations between circumcision status, circumcision age, and HIV. Circumcision age was dichotomised into men who were circumcised before or at age 12 years (pre-pubertal) and men circumcised at age 13 or older (post-pubertal). In a sub-analysis using data from the 2006 cohort, we determined the association between traditional circumcision vs. medical circumcision and the risk of HIV infection. The results are reported as crude and adjusted odds ratio (COR/AOR) with the corresponding 95% confidence intervals (CI).

**Ethics and consent:** The two cohort studies from which this secondary data analysis was performed were approved by the Guinea-Bissau Government Ethics Committee and the Danish Central Scientific Ethics Committee. All participants were counselled and gave informed verbal consent prior to HIV testing.

## Results

### Prevalence of circumcision

A total of 1,014 males from the 1996 cohort and 954 males from the 2006 cohort were included in this study. All of the major ethnic groups in Guinea Bissau are represented in our analysis and reflect the distribution in Bissau. In the 1996 cohort, 89% (n=904) were circumcised, and the prevalence of circumcision had increased to 93% (n=888) ten years later. A significantly higher proportion of the 2006 cohort were circumcised compared to the 1996 cohort among men aged 14-24 years (p-value=0.006) and men aged 35 and above (p-value=0.031). The overall prevalence of circumcision in both cohorts was above 80% for all ethnic groups except the Balanta (only 65% were circumcised in the 1996 cohort and 69% in the 2006 cohort). In both cohorts, the Balanta and Papel comprised over 70% of those uncircumcised. Muslims in both cohorts had a circumcision rate of approximately 99%. As expected, the prevalence of circumcision increased with age. A complete overview of the ethnic groups and other socio-demographic factors, circumcision status and HIV prevalence is shown in Table 1.

**Table 1:** HIV sero-prevalence by circumcision status, circumcision age and socio-demographic characteristics

	1996 cohort						2006 cohort				
		No. Men (% HIV-positive)						No. Men (% HIV-positive)			
	Total N=1014 (col%)			Age of circumcision			Total N=954 (col%)			Age of circumcision	
		Not Circumcised	Circumcised	≤12 years	≥13 years			Not Circumcised	Circumcised	≤12 years	≥13 years
**Age group**											
Age 14-24	401(39.6)	77(1.3)	324(0.9)	172(1.2)	152(0.7)		366(38.4)	46(0.0)	320(2.5)	118(1.7)	202(3.0)
Age 25-34	273(26.9)	15(20.0)	258(4.7)	136(4.4)	122(4.9)		296(31.0)	14(7.7)	282(4.2)	124(3.2)	158(5.1)
Age >35	340(33.5)	18(33.3)	322(12.7)	105(17.1)	217(10.6)		292(30.6)	7(57.1)	285(12.6)	108(16.7)	177(10.2)
**Ethnic group**											
Papel	388(38.3)	45(4.4)	343(4.1)	113(0.9)	230(5.7)		409(42.9)	14(0.0)	395(5.3)	105(8.6)	290(4.1)
Balanta	104(10.3)	36(11.1)	68(5.9)	13(7.7)	55(5.5)		113(11.8)	35(8.6)	78(1.3)	16(0.0)	62(1.6)
Manjaco/ Mancanha	249(24.6)	13(7.7)	236(8.9)	134(8.2)	102(9.8)		209(21.9)	7(0.0)	202(8.4)	97(5.2)	105(11.4)
Mandinga/ Fula	98(9.7)	1(0.0)	97(8.3)	61(11.5)	36(2.8)		102(10.7)	0(0.0)	102(5.9)	70(5.7)	32(6.3)
Others^A^	175(17.3)	15(20.0)	160(5.6)	92(6.5)	68(4.4)		121(12.7)	10(20.0)	111(9.9)	63(9.5)	48(10.4)
**Religion**											
Muslim	146(14.4)	2(0.0)	144(8.3)	86(10.5)	58(5.2)		141(14.8)	1(0.0)	140(9.3)	90(8.9)	50(10.0)
Non-muslim	868(85.6)	108(9.3)	760(5.8)	327(5.2)	433(6.2)		813(85.2)	65(7.7)	748(5.8)	261(6.1)	487(5.5)
**Marital status**											
Single	520(51.3)	81(3.7)	439(1.8)	232(1.7)	207(1.9)		586(61.4)	55(1.8)	531(3.2)	226(2.2)	305(3.9)
Married	450(44.4)	25(24.0)	425(10.1)	163(12.9)	262(8.4)		343(36.0)	11(36.4)	332(11.14)	117(15.4)	215(8.8)
Widowed	12(1.2)	0(0.0)	12(8.3)	6(0.0)	6(16.7)		10(1.0)	0(0.0)	10(10.0)	3(33.3)	7(0.0)
Divorced	32(3.2)	4(25.0)	28(14.3)	12(8.3)	16(18.8)		15(1.6)	0(0.0)	15(6.7)	5(0.0)	10(10.0)
**Education**^B^											
None	366(36.1)	39(12.8)	327(9.8)	126(11.9)	201(8.5)		189(19.8)	10(33.3)	179(7.8)	56(3.6)	123(9.8)
Schooling	648(63.9)	71(7.0)	577(4.2)	287(3.8)	290(4.5)		765(80.2)	57(3.5)	708(5.9)	294(7.5)	414(4.8)

A Remaining small ethnic groups and foreigners i.e. Cape Verdean, Senegalese and Guinean (Republic of Guinea).

B Over 4 years of school and speaks/reads basic Portuguese.

### Age of circumcision

The overall median age of circumcision (MAC) increased from 13 years in the 1996 cohort (IQR 10-17, range 1-53) to 14 years in the 2006 cohort (IQR 10-18, range 0-49). In both cohorts, the Papel and the Balanta had a notably higher MAC compared to the other ethnic groups. Table 2 shows the median age of circumcision by ethnic group. For the Balanta, only 34% in the 1996 cohort and 37% in the 2006 cohort were circumcised before the median age of sexual debut. We found that over 78% of the Manjaco, Mancanha, Fula and Mandinga were circumcised before age of sexual debut in both cohorts.

**Table 2:** Median age of circumcision according to ethnic group

	1996 cohort					2006 cohort			
	N (%)	Median	IQR^A^	Range		N (%)	Median	IQR	Range
**Ethnic group**									
Papel	343(37.9)	15	11-18	1-35		395(44.5)	16	12-19	0-47
Balanta	68(7.5)	19.5	14-30	3-53		78(8.8)	17.5	14-26	5-49
									
Manjaco/ Mancanha	236(26.1)	12	9-14	1-49		202(22.8)	13	10-15	3-49
Mandinga/ Fula	97(10.7)	12	10-14	5-24		102(11.5)	10	7-14	2-32
Others^B^	160(17.7)	12	8-15	1-37		111(12.5)	12	9-15	4-42
**Total**	904(100.0)	13	10-17	1-53		888(100.0)	14	10-18	0-49

A Interquartile range, IQR.

B Remaining small ethnic groups and foreigners i.e. Cape Verdean, Senegalese and Guinean (Republic of Guinea).

### HIV, ethnicity and religion

The overall HIV prevalence in the 1996 cohort was 6.5% (66/1014), and 67% (n=44) of those men were infected with HIV-2. In the 2006 cohort, we found an overall HIV prevalence of 6.4% (61/954). In contrast to the 1996 cohort, 41% (n=25) of the HIV infected men in the 2006 cohort were HIV-2 infected. In the 1996 cohort, the Manjaco/Mancanha had the highest HIV prevalence (8.8%), while the Papel had the lowest prevalence at 4.1%. Ten years later, the HIV prevalence among the Manjaco/Mancanha was still high (8.1%) and was only surpassed by a mixed group of foreigners and small ethnic groups in Guinea-Bissau (10.7%). Conversely, the HIV prevalence among the Papel remained among the lowest, with only 5.1% infected. We found that being Manjaco/Mancanha was a significant factor for HIV infection in the 1996 cohort (AOR 2.13 (95% CI 1.06-4.30). Over 90% of Mandinga/Fula were self-reported Muslims and comprised more than 65% of all Muslims in both cohorts (data not shown). In both cohorts, we found that Muslims had a higher prevalence of HIV infection than non-Muslims; the prevalence was 8.2% in Muslims vs. 6.2% in non-Muslims for the 1996 cohort (COR 1.35, 95% CI 0.71-2.57) and 9.2% in Muslims vs. 5.9% in non-Muslims for the 2006 cohort (COR 1.62, 95% CI 0.86-3.05). Table 3 shows HIV prevalence associated with circumcision status and other covariates for both cohorts.

**Table 3:** Univariate and multivariate analysis of HIV risk factors adjusted for socio-demographic factors A

	1996 cohort				2006 cohort			
Variable	Total N=1014 (%)	HIV+ n(%)	COR (95%CI)	AOR (95%CI)	Total N=954 (%)	HIV+ n(%)	COR (95%CI)	AOR (95%CI)
**Age group**								
Age 14-24	401(39.6)	4(1.0)	1	1	366(38.4)	8(2.2)	1	1
Age 25-34	273(26.9)	15(5.5)	5.77(1.89-17.58)	**4.75(1.35-16.73)**	296(31.0)	13(4.4)	2.06(0.84-5.03)	1.71(0.65-4.55)
Age >35	340(33.5)	47(13.8)	15.92(5.67-44.68)	**12.0(3.21-44.85)**	292(30.6)	40(13.7)	7.10(3.27-15.43)	**4.84(1.68-13.94)**
**Ethnic group**								
Papel	388(38.3)	16(4.1)	1	1	409(42.9)	21(5.1)	1	1
Balanta	104(10.3)	8(7.7)	1.94(0.81-4.66)	1.21(0.49-3.28)	113(11.8)	4(3.5)	0.68(0.23-2.02)	0.48(0.15-1.56)
Manjaco/ Mancanha	249(24.6)	22(8.8)	2.25(1.16-4.38)	**2.13(1.06-4.30)**	209(21.9)	17(8.1)	1.64(0.84-3.17)	1.55(0.77-3.09)
Mandinga/ Fula	98(9.7)	8(8.2)	2.07(0.86-4.98)	1.83(0.72-4.70)	102(10.7)	6(5.9)	1.15(0.45-2.94)	1.09(0.41-2.91)
Others	175(17.3)	12(6.9)	1.71(0.79-3.70)	1.61(0.72-4.70)	121(12.7)	13(10.7)	2.22(1.08-4.59)	1.77(0.82- 3.82)
**Marital status**								
Single	520(51.3)	11(2.1)	1	1	586(61.4)	18(3.1)	1	1
Married	450(44.4)	49(10.9)	5.65(2.90-11.0)	1.54(0.65-3.65)	343(36.0)	41(12.0)	4.28(2.42-7.59)	2.07(0.93-4.62)
Widowed	12(1.2)	1(8.3)	4.21(0.50-35.49)	1.09(0.12-10.26)	10(1.1)	1(10.0)	3.51(0.42-29.17)	1.64(0.18-15.15)
Divorced	32(3.2)	5(15.6)	8.57(2.78-26.41)	1.93(0.53-7.06)	15(1.6)	1(6.7)	2.25(0.28-18.08)	1.06(0.12-9.53)
**Education**								
None	366(36.1)	37(10.1)	1	1	189(19.8)	17(9.0)	1	1
Schooling	648(63.9)	29(4.5)	0.42(0.25-0.69)	0.87(0.48-1.58)	756(80.2)	44(5.8)	0.62(0.34-1.11)	1.11(0.58-2.10)
**History of STIs**								
No	747(73.7)	35(4.7)	1	1	869(91.1)	53(6.1)	1	1
Yes	267(26.3)	31(11.6)	2.67(1.61-4.43)	**1.92(1.12-3.29)**	85(8.9)	8(9.4)	1.60(0.73-3.49)	**2.39(1.03-5.53)**
**Age of circumcision**								
Not circumcised	110(10.9)	10(9.1)	1	1	66(6.9)	5(7.6)	1	1
Pre-pubertal(≤12)	413(40.7)	26(6.3)	0.67(0.31- 1.44)	**0.33(0.13-0.84)**	351(36.8)	24(6.8)	0.90(0.33-2.44)	0.32(0.09-1.04)
Post-pubertal(≥13)	491(48.4)	30(6.1)	0.65(0.31- 1.37)	**0.25(0.10-0.62)**	537(56.3)	32(6.0)	0.77(0.29-2.06)	**0.29(0.09-0.93)**

A Odds Rations are adjusted for age, ethnicity, marital status, education, history of STIs and circumcision age.

### Association between circumcision and HIV

In both cohorts, the prevalence of HIV was higher for uncircumcised compared to circumcised men, i.e., 9.1% vs. 6.2% in the 1996 cohort and 7.6% vs. 6.3% in the 2006 cohort. When adjusted for age, ethnicity, civil status, education and history of STIs, we found that circumcision was protective against HIV in the 1996 cohort and the 2006 cohort with OR's of 0.28 (95% CI 0.12-0.66; p=0.004) and 0.30 (95% CI 0.09-0.93; p=0.037), respectively.

### HIV and circumcision age

In the 1996 cohort, HIV prevalence was 6.3% for men circumcised at age ≤ 12 (pre-pubertal), 6.1% for men circumcised at age ≥13 (post-pubertal) and 9.1% among those uncircumcised. Ten years later, HIV prevalence was 6.8% for men circumcised at age ≤ 12 (pre-pubertal), 6.0% for men circumcised at age ≥13 (post-pubertal) and 7.6% among those uncircumcised. The highest risk reduction of HIV infection was associated with post-pubertal circumcision in both the 1996 cohort and the 2006 cohort models with AORs of 0.25 (95% CI, 0.10-0.62) and 0.29 (95% CI, 0.09-0.93), respectively. Table 3 shows the AORs for age of circumcision and risk of HIV. In a second model, we examined behaviour-related variables such as age groups, previous military duty, history of travel and history of STIs in both cohorts. In the 2006 cohort model we also had data to include condom use (ever) and alcohol use (Table 4). As with the first model, pre-pubertal and post-pubertal circumcision were more protective than non-circumcision in the 1996 cohort, with AORs of 0.40 (95% CI, 0.17-0.94) and 0.26 (95% CI, 0.11-0.60), respectively. Surprisingly, there was no longer a significant association between lack of circumcision and HIV status in the 2006 cohort. To examine whether post-pubertal circumcision provided higher protection against HIV compare to early circumcision, we conducted a sub-analysis comparing pre- and post-pubertal circumcision. When uncircumcised males were excluded from the analysis, there was no statistically significant difference between post-pubertal and pre-pubertal circumcision in the 1996 cohort (AOR 0.76; 95% CI, 0.42-1.39) or the 2006 cohort (AOR 0.98; 95% CI, 0.54-1.76). Furthermore, no statistically significant difference between the two groups was found in an adjusted analysis using behaviour related variables (data not shown).

**Table 4:** Univariate and multivariate analysis of HIV risk factors adjusted for behaviour -related variables A.

	1996 cohort				2006 cohort B			
Variable	Total N=1014 (%)	HIV+ n(%)	COR (95%CI)	AOR (95%CI)	Total N=954 (%)	HIV+ n(%)	COR (95%CI)	AOR (95%CI)
**Age group**								
Age 14-24	401(39.6)	4(1.0)	1	1	366(38.4)	8(2.2)	1	1
Age 25-34	273(26.9)	15(5.5)	**5.77(1.89-17.58)**	**6.52(2.06-20.66)**	296(31.0)	13(4.4)	2.06(0.84-5.03)	2.04(0.81-5.15)
Age >35	340(33.5)	47(13.8)	**15.92(5.67-44.68)**	**20.23(6.76-60.58)**	292(30.6)	40(13.7)	**7.10(3.27-15.43)**	**7.94(3.33-18.90)**
**Military**								
No	888(87.6)	50(5.6)	1	1	874(92.1)	51(5.8)	1	1
Yes	126(12.4)	16(12.7)	**2.44(1.34-4.43)**	1.07(0.56-2.07)	75(7.9)	10(13.3)	**2.48(1.20-5.12)**	1.29(0.59-2.81)
**Travel outside Bissau**								
No	670(66.1)	39(5.8)	1	1	698(73.2)	34(4.9)	1	1
Yes	344(33.9)	27(7.9)	1.38(0.83-2.29)	0.77(0.44-1.33)	254(26.6)	27(10.6)	**2.32(1.37-3.94)**	1.26(0.70-2.25)
**Alcohol use**								
No	NA	NA	NA	NA	441(46.2)	27(6.1)	1	1
Yes	NA	NA	NA	NA	513(53.8)	34(6.6)	1.09(0.65-1.83)	0.82(0.48-1.43)
**Ever used condom**								
Yes	NA	NA	NA	NA	514(53.9)	32(6.2)	1	1
No	NA	NA	NA	NA	440(46.1)	29(6.6)	1.10(0.63-1.79)	0.85(0.47-1.51)
**History of STIs**								
No	747(73.7)	35(4.7)	1	1	869(91.1)	53(6.1)	1	1
Yes	267(26.3)	31(11.6)	**2.67(1.61-4.43)**	**2.11(1.24-3.58)**	85(8.9)	8(9.4)	1.60(0.73-3.49)	2.04(0.89-4.71)
**Age of circumcision**								
Not circumcised	110(10.9)	10(9.1)	1	1	66(6.9)	5(7.6)	1	1
Pre-pubertal(≤12)	413(40.7)	26(6.3)	0.67(0.31-1.44)	**0.40(0.17-0.94)**	351(36.8)	24(6.8)	0.90(0.33-2.44)	0.45(0.15-1.34)
Post-pubertal(≥13)	491(48.4)	30(6.1)	0.65(0.31-1.37)	**0.26(0.11-0.60)**	537(56.3)	32(6.0)	0.77(0.29-2.06)	0.38(0.13-1.09)

NA, not available.

A Odds Rations are adjusted for age, previous military duty (Guinea/Portuguese), history of travel, history of STIs, circumcision age and in the 2006 cohort, condom-use (ever) and alcohol use.

B Five missing values regarding military duty and 2 missing values regarding travel.

### Method of circumcision

In a sub-analysis based on data from the 2006 cohort, we examined the relationship between method of circumcision and HIV prevalence. Of the 954 participants from the 2006 cohort, 888 were circumcised and thus included in the analysis. Seventy-six percent (n=678) of all participants included reported being circumcised traditionally. For all ethnic groups, there was a trend (according to age group) of an increasing number of males being circumcised medically. Among males aged 15-24 years, 40.0% were circumcised in a hospital compared to 24.6% in the 25-34 years group and 4.6% in the 35+ group. The HIV prevalence for men circumcised traditionally was 7.7% compared to 1.9% circumcised medically. Traditional method of circumcision tended to be correlated with increased HIV risk when adjusted for ethnicity and age (AOR 2.7; 95% CI: 0.91-8.12).

## Discussion

In this study, we examined the association between MC, age and method of circumcision and the risk of HIV in Bissau, Guinea Bissau. Our findings are in line with the literature that has shown that MC is protective against HIV [8–11]. However, our data did not show increased protection based on early circumcision, contrary to previous research [23]. Other studies have found an inconsistent and non-significant relationship between circumcision age and HIV status [24, 25]. A higher HIV prevalence among ethnic groups who practice early circumcision may imply ethnic differences in risk behaviour. Among ethnic groups in Guinea-Bissau, the foreskin is linked to a lack of cleanliness, and according to some tribes, was the main reason some women might feel sexual repulsion [17]. This may imply that those who were circumcised early may be more sexually desirable and therefore more sexually active. Among the Balanta, who practice late circumcision, sexual relations between an uncircumcised man and a virgin women was regarded as hazardous and could result in a disease with symptoms similar to HIV/AIDS, which may deter some men from engaging in sexual relations [17]. A study in Kenya found that a variation in sexual behaviour among different ethnic groups may contribute to the large variations in HIV prevalence [26].

We found a higher HIV prevalence among Muslims, of whom the majority were Mandinga/Fula. This was surprising, as Islam encourages circumcision early in life and generally before puberty, which should, theoretically, provide increased protection against HIV [23]. Previous studies have found that being Muslim correlated with a lower risk of HIV [27]. Polygamy is common in some Muslim ethnic groups in Guinea Bissau [28], which may explain the higher prevalence of HIV. A study in Uganda found that being in a polygamous marriage was a risk factor for HIV [29]. Concurrent sexual partnerships may increase the spread of HIV [30]. Gianelli et al. found a higher risk of HIV-1 among pregnant Mandinga women compared to pregnant Balanta living in Guinea-Bissau, suggesting that female excision, which is prevalent among Muslim ethnic groups, could explain the ethnic differences [28]. Another study in Guinea Bissau, which found a higher HIV-2 prevalence among the Muslim ethnic groups of Fula and Mandinga, suggested that cohort female circumcision of dozens of young females (age 8-12) using the same ceremonial knife my lead to the spread of HIV [31]. Male circumcision, though widely practiced in Guinea Bissau, is carried out at different ages. Previous research on male circumcision in Guinea Bissau has underlined the multiple and interconnected dimensions that exist between ethnic groups. While MC is generally carried out as a collective rite of passage and identity, the practice is affected by religious, spiritual, social, biomedical, aesthetic and cultural factors [17]. Our study found a higher risk reduction among males circumcised after puberty. While our analysis failed to provide an explanation for this, similar results were found in both cohorts. Kibira et al. found that men circumcised between ages 10-14 had the highest percentage (48%) participating in higher-risk sex compared to those aged 15-19 (34%), yet the reason for this was unclear [25]. Furthermore, a study from South Africa found a higher prevalence of HIV among black males who underwent pre-pubertal (<12) circumcision compared to those with post-pubertal circumcision [24].

Over 75% of males in this study reported being circumcised traditionally. We found traditional MC practice to be a risk factor for HIV, but this was not statistically significant. Other studies have found that adverse effects following traditional circumcisions can be frequent [32, 33]. Traditional circumcision in some parts of Guinea-Bissau is still conducted on large groups of males, commonly by blacksmiths or shoemakers, on a highly vascular organ, which inevitably presents a risk of HIV transmission [17]. Bailey et al. found that traditional circumcision was associated with slower healing, more swelling, laceration and scarring compared to medical circumcision [33]. In Uganda, enhanced sexual activity and first intercourse among adults and young men has been reported during traditional circumcision seasons [34]. Penile HIV shedding is significantly increased after MC until wound healing [35]. Therefore, sexual intercourse shortly after circumcision and before complete wound-healing could further undermine the benefits of MC and increase the spread of HIV [34].

While several plausible biological explanations for the reduced transmission of HIV among circumcised men have been proposed, including the thickness of the keratin layer, distribution and density of target cells, foreskin surface area, “wetness” under the foreskin and difference in microbiome, the literature remains inconclusive [36]. Circumcision appears to be protective against HIV, yet our findings suggest that ethnical, methodological and temporal factors continue to play an unclear role in this relationship. A review by Morris et al. has underlined the many benefits of neonatal circumcision, advocating that MC be integrated into existing health systems as part of postnatal care [18]. Male circumcision is strongly linked to ancient rites and rituals and remains a mode for men to maintain their traditions [17]. Instituting infant circumcision might present a challenge to individuals and cultures in which circumcision is an important part of coming-of-age ceremonies, as is the case in Guinea-Bissau. The circumcision message is now universal, yet if this message of risk reduction is not delivered clearly and with supportive interventions, some men may opt towards high risk sexual behaviour [37]. Traditionally, circumcised men who are aware of the protective effect have been shown to endorse risk compensation, have a perceived lower risk of HIV infection, and were more likely to report unprotected vaginal sex [38]. Furthermore, incorrect beliefs about MC among women may lead to less condom use and increased sexual risk behaviour with partners of unknown HIV sero-status [39]. In West African countries such as Guinea Bissau, efforts are needed to address issues of risk behaviours and identify cultural factors that may undermine the efficacy of MC.

Our findings should be considered in light of a number of limitations. This study was based on cross-sectional data and we are not able to determine the temporality of circumcision to HIV infection. The retrospective, observational manner of this study limits our ability to control in detail for confounding factors such as sexual behaviour. The number of male participants in the survey limited logistical models to include only socio-demographic and risk behaviour related variables for the univariate and multivariate analysis. Information on circumcision was self-reported and may or may not have been influenced by recall bias or social desirability in regards to when and where circumcision was conducted. We were unable to assess whether responses were influenced by the presence of male research assistants, peers, or the fear of being stigmatised. In addition, 428 males were excluded from the 1996 cohort due to missing data on circumcision, creating a selection bias.

## Conclusion

Male circumcision is highly prevalent in Guinea-Bissau, yet there exist ethnic variations in the methods and timing of circumcision. While circumcision is protective overall against HIV, our findings suggest that factors such as traditional circumcision and sexual behaviour may increase HIV infection risk. Ethnical, methodological and temporal factors continue to play an unclear role in the relationship between MC and HIV. This complex relationship merits further research, including an analysis of risk factors associated with male circumcision.

### What is known about this topic

- Randomized trials have demonstrated that male circumcision can reduce the heterosexual acquisition of HIV by 60%
- Guinea-Bissau is burdened by an epidemic of HIV-1 and HIV-2 despite high rates of male circumcision

### What this study adds

- Circumcision was protective against HIV, yet this study suggests that ethnic, methodological and temporal factors continue to play an unclear role in this relationship
- Pre-pubertal circumcision was not associated with increased protection against HIV compared to post-pubertal circumcision
- Traditional circumcision may increase the risk of HIV in Guinea-Bissau
